# Socioeconomic inequalities in cancer survival in England after the NHS cancer plan

**DOI:** 10.1038/sj.bjc.6605752

**Published:** 2010-06-29

**Authors:** B Rachet, L Ellis, C Maringe, T Chu, U Nur, M Quaresma, A Shah, S Walters, L Woods, D Forman, M P Coleman

**Affiliations:** 1 Cancer Research UK Cancer Survival Group, Non-Communicable Disease Epidemiology Unit, Department of Epidemiology and Population Health, London School of Hygiene and Tropical Medicine, Keppel Street, London WC1E 7HT, UK; 2 National Cancer Intelligence Network, Queens House, 55/56 Lincolns Inn Fields, London WC2A 3PX, UK; 3 Northern and Yorkshire Cancer Registry and Information Service, St James's Institute of Oncology, Bexley Wing, St James's University Hospital, Beckett Street, Leeds LS9 7TF, UK

**Keywords:** relative survival, deprivation, socioeconomic inequalities, health policy

## Abstract

**Background:**

Socioeconomic inequalities in survival were observed for many cancers in England during 1981–1999. The NHS Cancer Plan (2000) aimed to improve survival and reduce these inequalities. This study examines trends in the deprivation gap in cancer survival after implementation of the Plan.

**Materials and method:**

We examined relative survival among adults diagnosed with 1 of 21 common cancers in England during 1996–2006, followed up to 31 December 2007. Three periods were defined: 1996–2000 (before the Cancer Plan), 2001–2003 (initialisation) and 2004–2006 (implementation). We estimated the difference in survival between the most deprived and most affluent groups (deprivation gap) at 1 and 3 years after diagnosis, and the change in the deprivation gap both within and between these periods.

**Results:**

Survival improved for most cancers, but inequalities in survival were still wide for many cancers in 2006. Only the deprivation gap in 1-year survival narrowed slightly over time. A majority of the socioeconomic disparities in survival occurred soon after a cancer diagnosis, regardless of the cancer prognosis.

**Conclusion:**

The recently observed reduction in the deprivation gap was minor and limited to 1-year survival, suggesting that, so far, the Cancer Plan has little effect on those inequalities. Our findings highlight that earlier diagnosis and rapid access to optimal treatment should be ensured for all socioeconomic groups.

Socioeconomic inequalities in survival have been reported for most adult cancers in England and Wales. Among adults diagnosed during 1981–1990, 5-year survival for those living in the most deprived areas was significantly lower than for those in the most affluent areas for 44 of 47 different cancers (Coleman *et al*, 1999). During the 1990s, cancer survival improved significantly for almost all the common cancers. For many cancers, however, survival improved more for patients living in affluent areas than for those living in deprived areas. As a result, the deficit in survival between rich and poor (the ‘deprivation gap’) widened during this time (Coleman *et al*, 2004). Such observations suggest that deprived patients may not have benefited equally from advances in early diagnosis and treatment.

The NHS Cancer Plan for England was published in late 2000 (Department of Health, 2000). It was designed to improve prevention, early diagnosis and screening, and to provide optimal treatment for all patients, thus improving survival and quality of life. One of the main aims of the Cancer Plan was to tackle inequalities in cancer survival for people from deprived or less affluent backgrounds. Inequalities in cancer mortality under 75 years of age between the most deprived 20% of Local Authority Districts (Spearhead LADs) and England as a whole decreased by 11.3% between the baseline period 1995–1997 and 2004–2006, already more than the 6% reduction that had been targeted for 2009–2011 (Department of Health, 2003, 2007).

Overall survival for adults diagnosed with one of the common cancers in England up to 1999 remains lower than in many comparable European countries (Berrino *et al*, 2007), but it has improved against the highest survival in Europe, and the number of deaths within 5 years of survival that would be avoidable in England if the highest level of survival in Europe had been achieved decreased slightly during the 1990s (Abdel-Rahman *et al*, 2009). Recent observations also suggest a modest acceleration of the previous upward trend in short-term survival in England since implementation of the NHS Cancer Plan, for patients diagnosed during 2001–2006 and followed up to 2007 (Rachet *et al*, 2009). Given the longstanding inequalities in cancer survival in England since the 1970s, however, we have examined the effectiveness of the Cancer Plan in reducing socioeconomic inequalities in survival.

We used national data on all cancer patients diagnosed in England during the 11 years 1996–2006 and followed up to the end of 2007. We estimated the survival of patients diagnosed in three calendar periods, defined in relation to the NHS Cancer Plan: 1996–2000 (5 years; before the Cancer Plan), 2001–2003 (3 years; initialisation) and 2004–2006 (3 years; implementation). We examined relative survival in five categories of socioeconomic deprivation and the ‘deprivation gap’ in survival between the most deprived and most affluent groups. Changes in the deprivation gap between successive calendar periods before, during and after the introduction of the Cancer Plan provide a quantitative measure of any change in the survival deficit, and an early indication of whether the plan is achieving its goal of reducing inequalities in survival.

## Materials and methods

The data, study design and analytical tools have been described (Rachet *et al*, 2009). Briefly, we examined the data for all adults (15–99 years) diagnosed in England during 1996–2006 with 1 of 21 common primary malignant neoplasms that represent 90.7% of all cancers. In all, 6% of cases were excluded from analysis because the patient's survival was either zero or unknown (tumour registered from a death certificate only), and 1.4% because of unknown vital status or sex, duplicate registration, synchronous tumours, or invalid dates or date sequences. Patients who had a previous cancer of the same organ or tissue at any time since 1971 were also excluded. In all, we were able to include some 2.2 million cancer patients in the analyses, 92.5% of those eligible (Table 1).

The National Cancer Registry contains no information regarding the socioeconomic status of individual cancer patients. Instead, an ecological measure of deprivation was used, on the basis of the socioeconomic characteristics of the Lower Super-Output Area (LSOA) in which each patient was resident at the time of diagnosis. The income domain score of the Indices of Multiple Deprivation (IMD2004) was categorised into five groups by quintiles of the 34 378 LSOAs (mean population 1500) in England. Cancer patients were assigned to the deprivation category of their LSOA (from 1 ‘most affluent’ to 5 ‘most deprived’), using the postcode of residence at diagnosis and a combined historic file, covering the whole study period, of 2.1 million unique full postcodes, each linked to a complete set of contemporary geographic area codes.

### Statistical analysis

We estimated relative survival for each of the five deprivation categories, for each cancer and each sex, and by year or period of diagnosis. Relative survival is the ratio of the observed probability of survival and the probability that would have been expected if the cancer patients had only experienced the normal (background) mortality of the general population in which they live, given the same distribution of factors such as age, sex, geographical area, calendar period and deprivation. Relative survival is the standard approach to estimating population-based survival. It does not rely on accurate reporting of the cause of death, and it enables estimation of long-term survival from cancer, when competing causes of death become more important. It can be interpreted as survival from cancer after adjustment for other causes of death.

Background mortality was derived from population life tables. Mortality rates vary widely between socioeconomic groups and geographic regions in England, so we constructed life tables by single year of age (0–99 years), sex, deprivation category and Government Office Region for 1991, 2001 and 2005, using the mid-year population estimates and the mean annual number of death registrations during the 3 years centred on the index year. Death records were assigned to deprivation categories through the postcode and LSOA in the same way as the cancer cases, so that background mortality was precisely matched to the deprivation categories of the cancer patients. Linear interpolation between the census-based life tables was used to obtain life tables for other calendar years in the period 1996–2005 (Cancer Research UK Cancer Survival Group, 2004). Life tables for 2006 and 2007 could not be constructed, because the relevant data (deaths during 2007–2008) were unavailable. Life tables for 2005 were used for those years, without extrapolation.

To account for the rapid change in cancer-related mortality in the period immediately after a cancer diagnosis, survival probabilities were estimated at short (e.g., 1-month) intervals in the first few months after diagnosis, then at progressively longer intervals up to 10 years after diagnosis, using the maximum-likelihood approach for individual data (Estève *et al*, 1990). We report the cumulative probabilities of relative survival at 1 and 3 years after diagnosis.

All patients were followed up for at least 1 year, so the cohort approach was applied throughout to estimate year-on-year trends in 1-year survival. For longer-term survival, patients were grouped into three calendar periods of diagnosis (1996–2000, 2001–2003 and 2004–2006). For the first two periods, 3-year survival was derived from a cohort design. The shorter period of follow-up data available for patients diagnosed during 2004–2006 meant that only the complete approach was available for analysis. Short-term predictions of survival were made for patients diagnosed in 2007 using the hybrid approach (Brenner and Rachet, 2004).

A variance-weighted linear regression was fitted, incorporating all deprivation-specific relative survival estimates, thus allowing changes in survival to vary by deprivation group and calendar period. The deprivation gap in survival was quantified as the simple difference (%) between fitted relative survival in the most deprived and most affluent groups within each calendar period. A negative value indicates that survival in the most deprived group is lower than survival in the most affluent group. Similarly, a negative value for any change in the deprivation gap means that it widened between successive calendar periods. All analyses were carried out in Stata 10 (StataCorp, College Station, TX, USA), including relative survival analyses with the publicly available Stata programme *strel* (Cancer Research UK Cancer Survival Group, 2006).

## Results

### One-year relative survival

**Figure 1:**
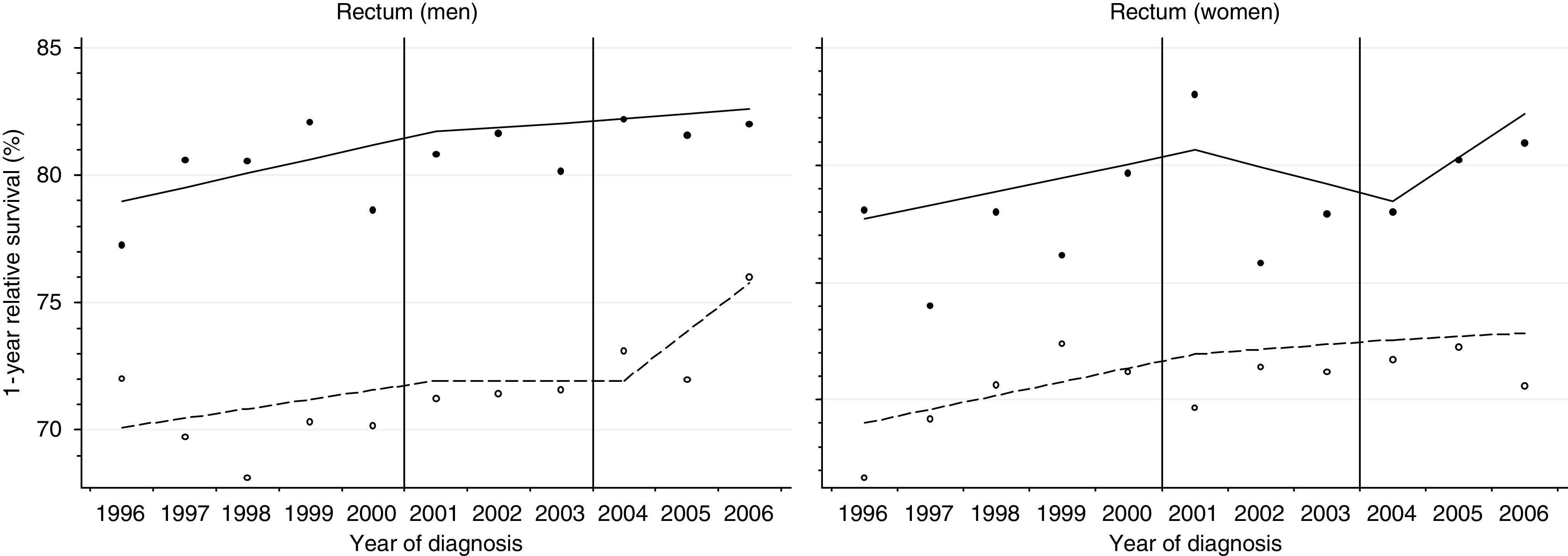
One-year relative survival for rectal cancer for the most deprived and most affluent groups, by sex, England 1996–2006. Lines are the regression plots fitted in a single model, which comprises every survival estimate by deprivation and calendar year (see text); dashed line: most deprived group; plain line: most affluent group.

Table 2 summarises the 1-year survival results for each cancer. As an example, Figure 1 shows the year-on-year variation in 1-year survival for rectal cancer among men and women in the most affluent and the most deprived groups in England. For men diagnosed with rectal cancer during 1996–2000 (before the plan), 1-year survival improved among both affluent and deprived groups, but more quickly for the affluent, and the deprivation gap widened (Figure 1; equivalent figures for each cancer in web-appendix). The rate of improvement slowed for men diagnosed during 2001–2003 (initialisation), especially for the most deprived, and the deprivation gap in 1-year survival widened further. For men diagnosed during 2004–2006 (implementation), 1-year survival rose more rapidly among the most deprived men than the most affluent, and the deprivation gap narrowed. One-year survival among all men in England rose from 79.0% in 1996 to 82.6% in 2006, and the deprivation gap narrowed from –8.9% to –6.8% over this period (Table 2). The gap widened slightly by −0.1% a year during 1996–2000 and 2001–2003 but narrowed by 1.1% a year during 2004–2006. Among women, the reduction in deprivation gap during 2001–2003 reflects a transient reduction in 1-year survival among the most affluent group.

We summarise here the detailed results for 17 cancers in men and 18 in women, 35 cancer-sex combinations in all (Table 2). Results are presented for three cancers in men only (larynx, prostate and testis) and for four cancers in women only (breast, cervix, uterus and ovary).

For patients diagnosed in 1996, 1-year survival was lower in the most deprived group than in the most affluent group (negative deprivation gap) for 34 of the 35 cancer-sex combinations, and for 27 of these, the deprivation gap was statistically significant at 5% (Table 2). Survival from brain tumours was slightly higher among men in the most deprived group (+0.6%). For patients diagnosed in 2006, the deprivation gap in 1-year survival was again negative for all but three cancer-sex combinations: Hodgkin's disease in men (+7.4%, *P*<0.05) and women (+2.0%) and myeloma in women (+0.4%).

For patients diagnosed during 1996–2000 (before the Cancer Plan), the deprivation gap narrowed (positive annual change) for five cancers in men and eight in women, but no reduction was more rapid than 1% per year (Table 2). The deprivation gap widened (negative annual change) for most of the 22 remaining cancer-sex combinations. The annual change was >−1% a year for cancers of the oesophagus (women) and ovary, and >−0.5% for four cancers in men and one in women.

For 2001–2003, the deprivation gap in 1-year survival narrowed for 11 of 17 cancers in men and 11 of 18 cancers in women: the rate of improvement was 1% or more per year for laryngeal cancer and Hodgkin's disease in men, and for malignancies of the ovary and brain in women. For other cancers, the deprivation gap was unchanged or became wider, but only for non-Hodgkin lymphoma in women did the gap widen by >0.5% a year.

For 2004–2006, the period beginning 3 years after implementation of the Cancer Plan, inequalities in 1-year survival narrowed for seven cancers in men and 12 in women, and for six malignancies the deprivation gap decreased by 1% or more a year: rectum, brain and Hodgkin's disease in men and pancreas, non-Hodgkin lymphoma and the leukaemias in women.

Overall, between 1996 and 2006, the deprivation gap in 1-year survival narrowed for 8 cancers in men and 13 in women, but widened for 9 cancers in men and 5 in women.

For four malignancies in women (stomach, ovary, myeloma and the leukaemias) and for Hodgkin's disease in men, the deprivation gap in 1-year survival decreased slightly but steadily during both the 3-year periods we had designated after introduction of the NHS Cancer Plan (2000–2003, 2004–2006). For three other malignancies in women (breast, kidney and melanoma) and for non-Hodgkin lymphoma in men, the deprivation gap decreased slightly but consistently over the entire period 1996–2006. A majority of the larger falls in the deprivation gap in any given period coincided with a decrease or a plateau in the trend in survival among the most affluent patients: Hodgkin's and non-Hodgkin lymphoma in both sexes and brain tumours in women (web-figures).

**Figure 2:**
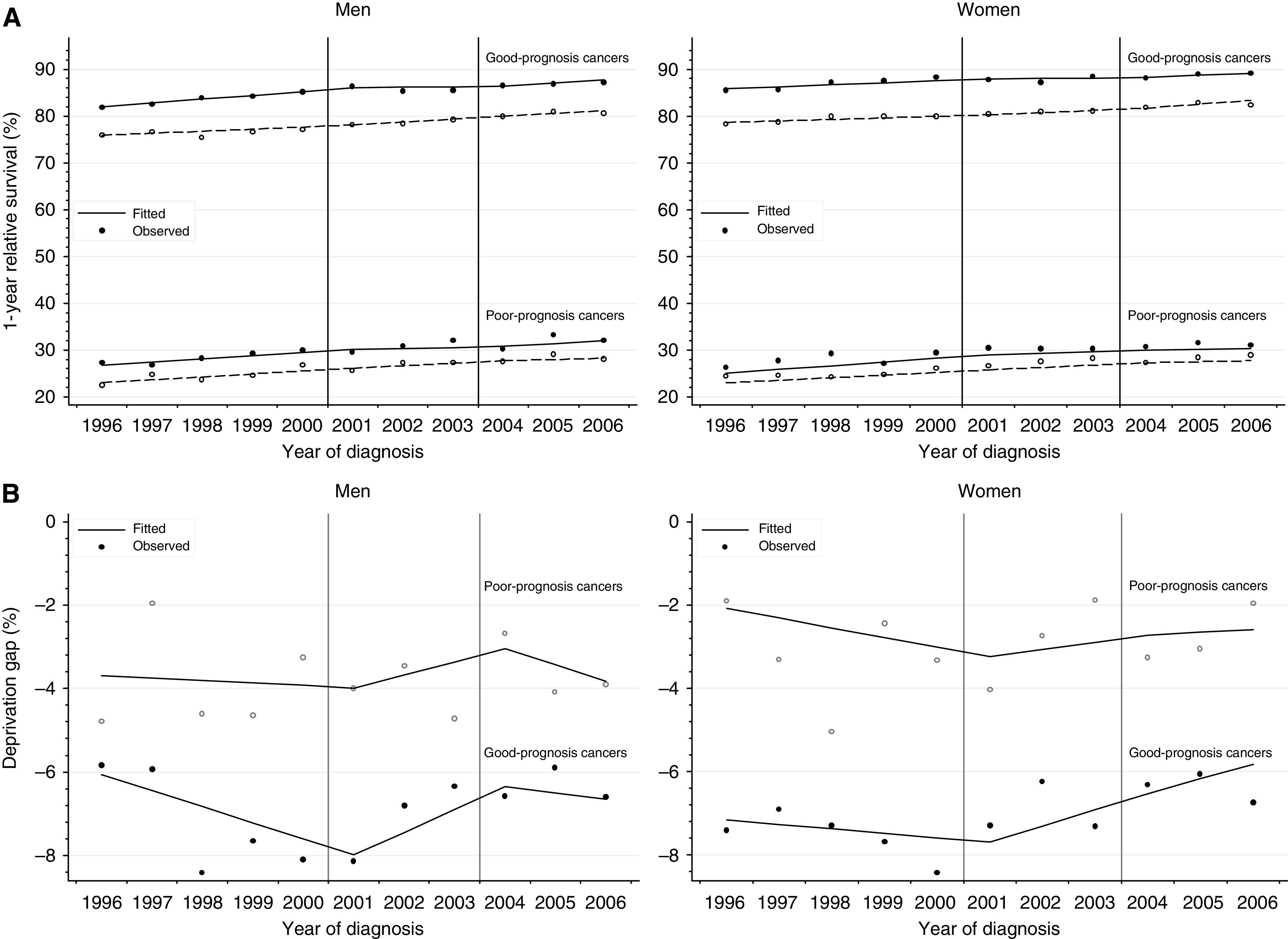
(**A**) Trends in 1-year relative survival for the most deprived and most affluent groups, by cancer prognosis, England 1996–2006. Lines are the regression plots fitted in a single model, which comprises every survival estimate by deprivation and calendar year (see text); dashed line: most deprived group; plain line: most affluent group. (**B**) Trends in deprivation gap in 1-year relative survival, by cancer prognosis, England 1996–2006. Deprivation gap is the simple difference in 1-year relative survival between the most deprived group and the most affluent group. Lines are the regression plots fitted in a single model, which comprises every survival estimate by deprivation and calendar year (see text).

We chose to summarise trends in short-term survival and in the deprivation gap in survival for two broad groups of malignancies, defined by 1-year relative survival in England in 1996 of <40% (‘poor prognosis’ group: oesophagus, stomach, pancreas, lung and brain) or >60% (‘good prognosis’ group: the other 16 cancers). The patients in each group were analysed together, not separated by site or type of cancer. One-year survival trends were estimated for good-prognosis and poor-prognosis cancers, by sex, deprivation and year of diagnosis. Overall, 1-year survival rose in both sexes, both for good-prognosis and poor-prognosis cancers, within each of the three periods 1996–2000, 2001–2003 and 2004–2006, and for all deprivation categories (Table 3, Figure 2A: only the most affluent and the most deprived groups are plotted).

For cancers with a good prognosis, the deprivation gap during 2004–2006 was around −6 to −8% in both sexes. This is wider than for cancers with a poor prognosis, around −2 to −3% (Figure 2B). Both for good-prognosis and poor-prognosis cancers, the deprivation gap in 1-year survival widened during 1996–2000, but then narrowed during 2001–2003 (Figure 2B). For good-prognosis cancers in women, the deprivation gap in 1-year survival continued to narrow more quickly during 2004–2006, but it was stable for poor-prognosis cancers. For men, no obvious pattern was observed in 2004–2006, for both good-prognosis and poor-prognosis cancers.

Patterns of 1-year survival by deprivation and over time were also analysed with non-linear regression models, using cubic regression splines. In spite of some differences for the less common cancers, the overall picture changed very little, and the conclusions were identical.

### Three-year relative survival

A significant deficit in 3-year survival among patients in the more deprived groups was observed for 33 of the 35 cancer-sex combinations examined during 1996–2000, for 26 in 2001–2003 and for 24 among patients diagnosed during 2004–2006 (web-table, equivalent to Table 2). Only for brain tumours in men was the deprivation gap in 3-year survival consistently positive (higher survival in more deprived groups) throughout the period 1996–2006. Short-term predictions of 3-year survival for patients diagnosed in 2007 suggest that the number of cancer-sex combinations with a significant deprivation gap will decrease further to 18 of 35. However, these changes mainly reflect a decrease in statistical power rather than a narrowing of the deprivation gap in survival, because the data sets for 2001–2003 and 2004–2006 (3 years) are 40% smaller than for 1996–2000 (5 years). The overwhelming pattern of lower survival in more deprived groups for almost all cancers seems more persuasive than whether individual estimates of the deprivation gap are statistically significant. Thus, a negative deprivation gap in 3-year survival was observed for 34 of 35 cancer-sex combinations in 1996–1999 and for 31 in 2004–2006, and is predicted for 28 of 35 cancer-sex combinations for patients diagnosed in 2007. Only for a few cancers did the deprivation gap itself actually narrow over time, confirming the overall lack of improvement.

We also examined changes over time in the deprivation gap in 3-year survival for cancers that we defined as having a ‘good’ or ‘poor’ 1-year prognosis (60% or above and under 40%, respectively, for patients diagnosed in England in 1996). There was no obvious pattern in either group (Table 3). In particular, in spite of the expected ceiling effect for cancers for which survival was over 80%, the narrowing of the deprivation gap was not more marked for cancers with a good prognosis than for those with a poor prognosis.

### Excess hazard of death

**Figure 3:**
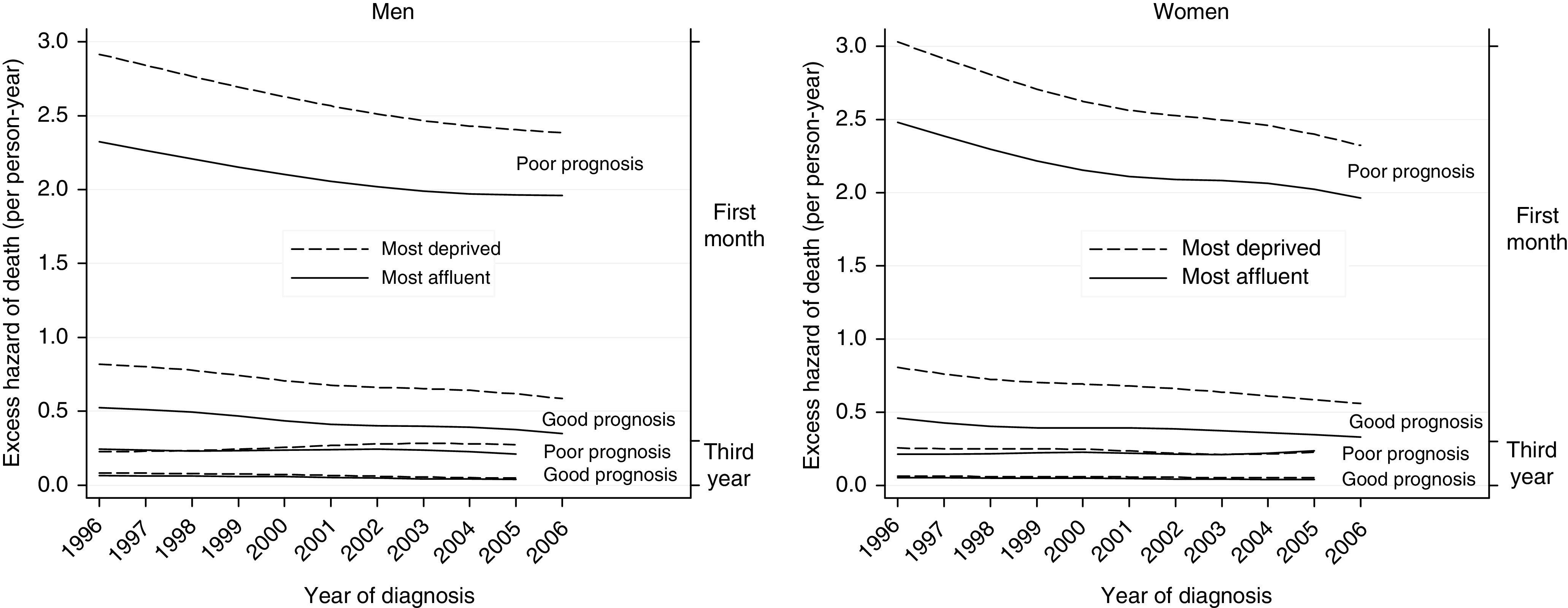
Excess hazard of death for the most deprived and most affluent groups, by cancer prognosis, England 1996–2006.

The excess hazard of death among cancer patients, relative to the general population, is naturally much higher in the first month after diagnosis than in the third year after diagnosis, and much higher for cancers with a ‘poor’ 1-year prognosis than for those with a ‘good’ prognosis (Figure 3). The excess hazard is also much higher for patients in the most deprived group than for those in the most affluent group. Strikingly, however, while there is a large socioeconomic difference in the excess hazard of death in the period shortly after diagnosis, as exemplified for the first month in Figure 3, no such difference in the excess hazard is observed during the third year after diagnosis.

This pattern was present throughout the period 1996–2006, in both sexes. The excess hazard of death decreased steadily both for affluent and deprived patients, and for cancers with both ‘good’ and ‘poor’ prognosis, but these trends were not accompanied by a narrowing of the deprivation gap.

## Discussion

This study updates the effect of socioeconomic deprivation on cancer survival in England to the end of 2007. It includes over 2 million patients diagnosed with one of 21 common cancers in the 11-year period spanning the introduction of the first comprehensive cancer plan in 2000.

Survival for patients in the most deprived group was significantly lower than among the most affluent patients for most cancers in both sexes, extending observations made for patients diagnosed during 1971–1990 (Coleman *et al*, 1999) and 1986–1999 (Coleman *et al*, 2004). The overall pattern of lower survival in the more deprived groups has changed very little over the three successive calendar periods that we defined *a priori* in relation to the NHS Cancer Plan. The socioeconomic gradient in 1-year survival often remained the same, or even widened for a few cancers, although the gap narrowed for several cancers of good prognosis.

Socioeconomic inequalities in cancer survival in England and Wales are well documented. Similar disparities have been reported in Scotland (Shack *et al*, 2007), Denmark (Dalton *et al*, 2008), the United States and, to a lesser extent, in Canada (Gorey *et al*, 2009), and in other countries (Kogevinas and Porta, 1997; Woods *et al*, 2006). With a few exceptions, most of the socioeconomic deficits in survival occur shortly after diagnosis, and they tend to attenuate or disappear with time since diagnosis (Rachet *et al*, 2008). The findings reported here confirm persistent and wide socioeconomic inequalities in the excess hazard of death in the period immediately after a cancer diagnosis. They indicate that more attention should be given to earlier diagnosis and prompt access to optimal treatment for all socioeconomic groups. In 2003, the *Tackling Health Inequalities* strategy set out to improve access to cancer services by ensuring that, by 2005, all cancer patients were treated within a month of diagnosis and within 2 months of urgent referral (Department of Health, 2003), thus reaffirming the waiting time targets initialised in the Cancer Plan. Earlier diagnosis also became a central goal of the *National Awareness and Early Diagnosis Initiative* in 2008 (Richards, 2009), which seeks to raise symptom awareness and encourage early presentation.

The origins of the deprivation gap in cancer survival are still not clear: the roles played by tumour, patient and health-care system factors, respectively, remain controversial (Woods *et al*, 2006; Rachet *et al*, 2008). The inequalities are not simply explained by more advanced stage at diagnosis among deprived groups (Schrijvers *et al*, 1995; Jeffreys *et al*, 2006) or, for colorectal cancers at least, by more frequent or more severe co-morbidity (Shack, 2009).

Socioeconomic differences in diagnosis, investigation and treatments have also been identified in the United Kingdom, both qualitatively and quantitatively (Dixon *et al*, 2003). In the United States, both socioeconomic and ethnic disparities in cancer survival have been reported (Singh *et al*, 2004). Ethnicity, often considered a proxy for deprivation, is strongly associated with the type of health insurance (McDavid *et al*, 2003). The survival deficit for colorectal cancer among US Blacks was not apparent when health care was provided within the equal-access Veterans Administration system (Rabeneck *et al*, 2003). It is more surprising that inequalities in access to the health-care system have been reported within universal-access health-care systems, whether centrally funded, as in the United Kingdom, or insurance based. For example, in Canada, lower socioeconomic status was associated with higher mortality after stroke and both reduced access to some health services (Kapral *et al*, 2002) and treatment at low-volume hospitals (Saposnik *et al*, 2008). In England, a population-based study showed that deprived patients with rectal cancer were more likely than affluent patients to undergo abdomino-perineal resection rather than anterior resection, even after controlling for Duke's stage (Morris *et al*, 2008). By contrast, no socioeconomic disparity in colorectal cancer survival was observed among patients in a large clinical trial in which, by definition, every patient of a given arm received the same treatment (Nur *et al*, 2008).

These strands of evidence suggest that health-care system factors do have a major role in inequalities in survival, over and above tumour and patient factors.

The NHS Cancer Plan of 2000 may have led to quickening improvements in cancer survival (Rachet *et al*, 2009), but we have not yet found strong evidence that implementation of the Cancer Plan has been followed by a reduction in the deprivation gap in survival. Without continuing actions targeted at lower socioeconomic groups, however, the inequalities may even become wider, because survival will increase more rapidly in affluent groups (Coleman *et al*, 2004). The greater involvement of general practitioners in the early detection of cancer may help reduce these inequalities (Anon, 2009). As set out in the Calman–Hine report (Expert Advisory Group on Cancer, 1995; Haward, 2006) in 1995, reducing the geographical and socioeconomic disparities in outcome still requires action to extend the availability of optimal health care for all, and to ensure that all patients seek and obtain access to those services at the earliest possible moment. Further analyses including more detailed information on the cancers and their management are essential for understanding the recent and future patterns in the socioeconomic inequalities in cancer survival. Such studies have recently become possible since the linkage between the National Cancer Registry data and Hospital Episode Statistics data.

## Figures and Tables

**Table 1:** Number of patients eligible for survival analysis, exclusions (%) and no. (%) of patients included: 21 common cancers, England, adults (15–99 years) diagnosed 1996–2006 and followed up to 2007

		**Exclusions**			**Patients included**	
**Malignancy**	**Eligible for analyses**	**Death certificate only^a^**	**Zero survival^b^**	**Other^c^**	**No.**	**%**
*Oesophagus*	66 479	4.0	1.8	0.5	62 359	93.8
*Stomach*	82 101	4.9	2.6	0.5	75 467	91.9
*Colon*	199 149	4.5	2.4	1.6	182 244	91.5
*Rectum*	117 410	2.4	1.3	0.6	112 319	95.7
*Pancreas*	66 322	11.1	5.1	0.4	55 327	83.4
*Larynx (M)*	16 154	2.1	0.8	0.8	15 537	96.2
*Lung*	343 065	7.0	3.8	0.8	303 422	88.4
*Melanoma*	69 921	1.0	0.2	1.6	67 963	97.2
*Breast (F)*	386 627	2.2	0.8	3.6	361 105	93.4
*Cervix*	27 219	1.7	0.8	1.2	26 200	96.3
*Uterus*	55 579	2.2	0.9	0.7	53 484	96.2
*Ovary*	61 986	4.7	2.2	0.7	57 253	92.4
*Prostate*	280 790	3.4	1.1	0.9	265 753	94.6
*Testis*	17 683	0.4	0.2	2.2	17 210	97.3
*Kidney*	59 381	5.7	3.1	0.9	53 609	90.3
*Bladder*	102 927	2.9	1.1	0.9	97 908	95.1
*Brain*	36 630	4.9	2.0	1.0	33 717	92.0
*Hodgkin′s disease*	12 880	0.8	0.6	1.2	12 548	97.4
*Non-Hodgkin lymphoma*	87 685	3.1	2.1	1.2	82 082	93.6
*Myeloma*	34 379	5.1	2.3	0.7	31 593	91.9
*Leukaemia*	62 197	7.2	3.1	0.8	55 260	88.8
*Total*	2 186 564	4.1	2.0	1.4	2 022 360	92.5

a Registration from a death certificate only.

b Date of diagnosis same as date of death, but record not flagged as a ‘death certificate only’ registration.

c Aged 100 years or over at diagnosis, vital status or sex unknown, sex-site error, invalid dates, duplicate registration, synchronous tumours or a previous cancer of the same organ or tissue at some time since 1971.

**Table 2:** Deprivation gap in 1-year relative survival (%) by sex in 1996 and 2006, and annual change in the deprivation gap in survival within period of diagnosis: adults (15–99 years) diagnosed 1996–2006 and followed up to 2007, 21 common cancers, England

					**Calendar period of diagnosis (NHS Cancer Plan periodisation)**					
					**Period 1**		**Period 2**		**Period 3**	
	**1996**		**2006**		**1996–2000 (Pre-Cancer Plan)**		**2001–2003 (Initialisation)**		**2004–2006 (Implementation)**	
**Malignancy**	**Survival in most affluent**	**Deprivation gap (%)^a^**	**Survival in most affluent**	**Deprivation gap (%)^a^**	**Annual change in deprivation gap^b^**	**95% CI**	**Annual change in deprivation gap^b^**	**95% CI**	**Annual change in deprivation gap^b^**	**95% CI**
*Oesophagus*										
Men	30.0	−4.8^*^	43.9	−8.4^**^	−0.4	−1.2, 0.4	0.0	−1.1, 1.1	−0.3	−2.3, 1.6
Women	25.3	−0.9	38.2	−7.4^*^	−1.2	−2.2, −0.1	0.9	−0.6, 2.4	−1.1	−3.7, 1.6
										
*Stomach*										
Men	34.9	−5.0^**^	43.3	−4.4^*^	0.0	−0.8, 0.7	−0.1	−1.2, 1.0	0.4	−1.5, 2.4
Women	33.6	−4.8^*^	40.4	−3.7	−0.2	−1.2, 0.8	0.2	−1.2, 1.7	0.5	−2.2, 3.2
										
*Colon*										
Men	72.1	−8.0^**^	76.6	−7.7^**^	0.2	−0.4, 0.8	−0.3	−1.1, 0.4	0.2	−1.1, 1.4
Women	69.2	−7.2^**^	75.4	−10.6^**^	−0.2	−0.8, 0.4	−0.2	−1.0, 0.6	−0.4	−1.7, 0.9
										
*Rectum*										
Men	79.0	−8.9^**^	82.6	−6.8^**^	−0.1	−0.8, 0.5	−0.1	−0.9, 0.7	1.1	−0.2, 2.5
Women	77.7	−8.7^**^	82.2	−9.4^**^	0.0	−0.8, 0.8	0.6	−0.4, 1.7	−1.1	−2.9, 0.6
										
*Pancreas*										
Men	14.9	−2.5	19.4	−4.9^*^	−0.7	−1.4, 0.1	0.7	−0.3, 1.7	−0.4	−2.1, 1.4
Women	14.2	−3.9^*^	17.5	−2.6	0.1	−0.6, 0.8	−0.5	−1.5, 0.5	1.0	−0.8, 2.7
										
*Larynx*										
Men	89.2	−6.6^*^	90.0	−7.4^*^	−0.5	−1.6, 0.5	1.2	−0.2, 2.6	−1.0	−3.5, 1.5
										
*Lung*										
Men	24.8	−3.3^**^	27.4	−1.6	0.2	−0.2, 0.5	0.3	−0.2, 0.8	−0.3	−1.1, 0.6
Women	24.7	−1.5	30.9	−3.1^*^	−0.2	−0.6, 0.3	−0.1	−0.8, 0.5	0.0	−1.0, 1.1
										
*Melanoma*										
Men	95.6	−3.5^*^	97.1	−2.9^*^	−0.1	−0.8, 0.5	0.6	−0.2, 1.3	−0.3	−1.5, 0.8
Women	97.8	−1.4	98.0	−0.4	0.0	−0.4, 0.4	0.1	−0.4, 0.6	0.2	−0.5, 1.0
										
*Breast*										
Women	95.8	−4.0^**^	97.8	−2.6^**^	0.1	0.0, 0.3	0.1	−0.1, 0.3	0.1	−0.2, 0.4
										
*Cervix*										
Women	88.9	−7.7^**^	90.3	−6.0^*^	0.4	−0.3, 1.1	−0.3	−1.4, 0.7	0.3	−1.6, 2.1
										
*Uterus*										
Women	88.9	−4.1^**^	92.8	−3.8^*^	−0.4	−1.0, 0.2	0.7	0.0, 1.4	−0.1	−1.2, 1.1
										
*Ovary*										
Women	67.9	−4.6^*^	71.9	−3.4	−1.0	−1.7, −0.3	1.1	0.2, 2.1	0.8	−0.8, 2.5
										
*Prostate*										
Men	89.6	−3.6^**^	97.0	−2.9^**^	−0.2	−0.5, 0.0	0.6	0.3, 0.9	−0.2	−0.7, 0.2
										
*Testis*										
Men	98.9	−1.5	99.5	−1.7^*^	0.2	−0.2, 0.6	−0.5	−1.0, 0.1	0.2	−0.7, 1.1
										
*Kidney*										
Men	68.0	−6.9^**^	71.8	−7.0^*^	−0.1	−1.0, 0.9	0.3	−0.9, 1.5	−0.3	−2.3, 1.8
Women	63.8	−6.6^*^	70.9	−4.2	0.2	−1.0, 1.5	0.0	−1.6, 1.6	0.3	−2.3, 3.0
										
*Bladder*										
Men	86.1	−7.0^**^	80.2	−7.1^**^	0.0	−0.5, 0.6	0.7	−0.2, 1.5	−1.1	−2.7, 0.4
Women	77.1	−9.9^*^	71.6	−14.2^**^	0.2	−0.8, 1.3	0.0	−1.5, 1.5	−2.0	−4.6, 0.6
										
*Brain*										
Men	30.2	0.6	36.4	−1.5	−0.6	−1.7, 0.6	−0.4	−2.0, 1.1	1.1	−1.6, 3.8
Women	31.6	−7.6^*^	30.6	−1.0	0.4	−0.9, 1.8	1.0	−0.8, 2.9	−0.2	−3.4, 2.9
										
*Hodgkin′s disease*										
Men	92.8	−1.3	86.5	7.4^*^	−0.6	−1.8, 0.6	1.1	−0.7, 2.9	2.6	−0.6, 5.8
Women	94.7	−6.1^*^	86.4	2.0	0.9	−0.3, 2.2	−0.1	−2.0, 1.7	0.9	−2.7, 4.5
										
*Non-hodgkin lymphoma*										
Men	73.2	−7.8^**^	77.6	−5.7^*^	0.2	−0.6, 1.0	0.2	−0.9, 1.2	0.1	−1.6, 1.8
Women	75.0	−10.7^**^	77.7	−4.3^*^	0.5	−0.3, 1.4	−0.7	−1.8, 0.4	2.0	0.2, 3.9
										
*Myeloma*										
Men	67.1	−6.1^*^	71.5	−9.0^*^	−0.2	−1.5, 1.2	−0.1	−1.8, 1.7	−0.5	−3.6, 2.5
Women	64.0	−4.2	68.5	0.4	−0.2	−1.6, 1.3	0.0	−1.9, 2.0	1.9	−1.5, 5.2
										
*Leukaemia*										
Men	66.9	−4.2^*^	65.2	−6.6^*^	−0.5	−1.5, 0.5	0.2	−1.1, 1.5	−0.1	−2.4, 2.2
Women	63.1	−5.1^*^	61.1	−1.2	−0.6	−1.8, 0.5	0.7	−0.8, 2.3	1.5	−1.3, 4.2

Abbreviation: CI=confidence interval.

a Absolute difference (%) between relative survival in the most deprived and the most affluent groups, derived from variance-weighted regression. A negative value means that survival in the most deprived group is lower than survival in the most affluent group.

b Mean absolute change (%) in the deprivation gap in survival within the period. A neqative value means that the deprivation gap has widened.

^*^P<0.05; ^**^P<0.001.

**Table 3:** Deprivation gap in 1-year and 3-year relative survival (%), by sex and calendar period of diagnosis, and trends in deprivation gap (%) in survival, adults (15–99 years) diagnosed 1996–2006 and followed up to 2007: cancers with ‘good’ and ‘poor’ prognosis, England

	**Calendar period of diagnosis (NHS Cancer Plan periodisation)**											
	**Period 1**		**Period 2**		**Period 3**							
	**1996–2000 (Pre-Cancer Plan)**		**2001–2003 (Initialisation)**		**2004–2006 (Implementation)**		**Change in deprivation gap since previous period^b^**					
**Time since diagnosis**	**Survival in most affluent**	**Deprivation gap (%)^a^**	**Survival in most affluent**	**Deprivation gap (%)^a^**	**Survival in most affluent**	**Deprivation gap (%)^a^**	**2001–2003**	**95% CI**		**2004–2006**	**95% CI**	
*Prognostic group*												
*One year*												
Good prognosis^c^												
Men	85.2	−7.6^**^	86.2	−6.9^**^	87.7	−6.6^**^	0.7	0.1,	1.3	0.3	−0.7,	1.2
Women	87.5	−7.6^**^	88.1	−6.9^**^	89.1	−5.8^**^	0.7	0.1,	1.2	1.1	0.2,	2.0
Poor prognosis^d^												
Men	29.4	−3.9^**^	30.5	−3.4^**^	32.0	−3.8^**^	0.6	−0.4,	1.6	−0.5	−2.2,	1.3
Women	28.2	−3.0^**^	29.7	−2.9^**^	30.3	−2.6^*^	0.1	−1.1,	1.3	0.3	−1.8.	2.4
												
*Three years*												
Good prognosis^c^												
Men	69.3	−8.6^**^	73.4	−9.4^**^	75.9	−8.8^**^	−0.7	−1.6,	0.2	0.5	−0.5,	1.5
Women	74.2	−9.1^**^	76.2	−9.0^**^	78.0	−7.6^**^	0.1	−0.6,	0.9	1.3	0.4,	2.2
Poor prognosis^d^												
Men	11.0	−1.9^**^	11.8	−2.0^**^	12.2	−1.7^**^	−0.1	−0.9,	0.7	0.3	−0.7,	1.3
Women	11.1	−1.8^**^	12.0	−1.2^*^	11.9	−0.8	0.6	−0.4,	1.6	0.4	−0.8,	1.7

Abbreviation: CI=confidence interval.

a Absolute difference (%) between relative survival in the most deprived and most affluent groups, derived from variance-weighted regression. A negative value means that survival in the most deprived group is lower than survival in the most affluent group.

b Mean absolute change (%) in the deprivation gap in survival since the previous period. A neqative value means that the deprivation gap has widened.

c ‘Good-prognosis’ cancers are: bladder, breast, cervix, colon, Hodgkin's disease, kidney, larynx, leukaemia, melanoma, myeloma, Non-Hodgkin lymphoma, ovary, prostate, rectum, testis and uterus.

d ‘Poor-prognosis’ cancers are: brain, lung, oesophagus, pancreas and stomach. ^*^P<0.05; ^**^P<0.001.
