# Supplementary material for: Socioeconomic inequalities in cancer survival in England after the NHS cancer plan
Source: Br J Cancer. 2010 Jun 29;103(4):446–53. doi: 10.1038/sj.bjc.6605752 (PMC2939774; doi:10.1038/sj.bjc.6605752)
Supplement: Supplementary Figure and Table [file 6605752x1.pdf]

Figures 1

One-year relative survival for the most deprived and most affluent groups, by sex, England 1996-2006

Footnote to Figures 1:
Lines are the regression plots fitted in a single model (see text); dashed line: most deprived group; plain line: most affluent group

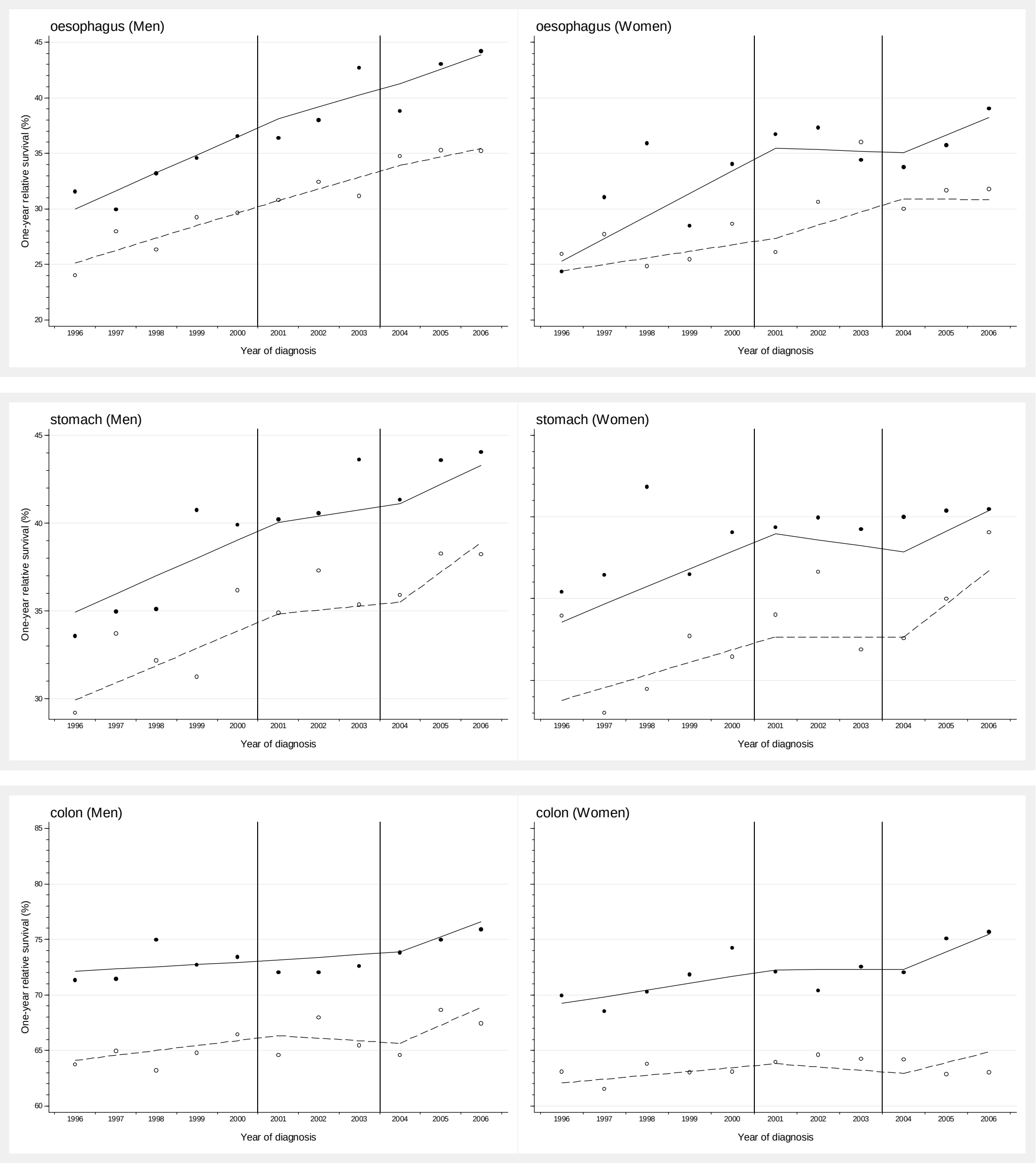

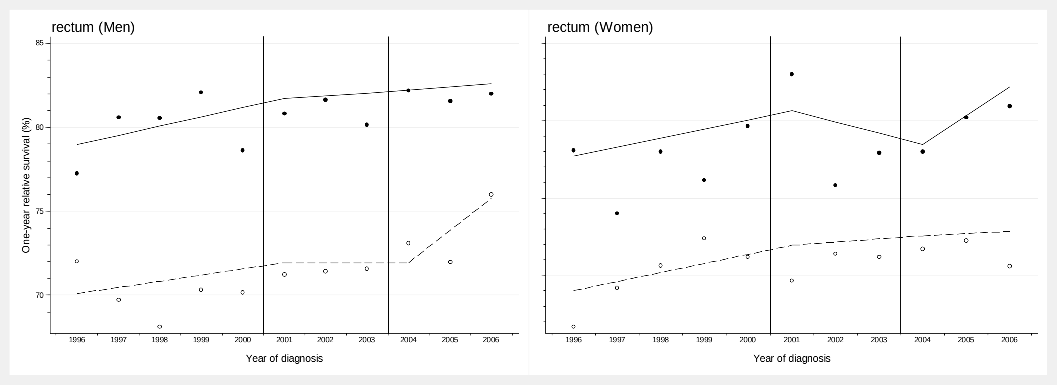
rectum (Men)
rectum (Women)
One-year relative survival (%)
Year of diagnosis
85
80
75
70
1996
1997
1998
1999
2000
2001
2002
2003
2004
2005
2006

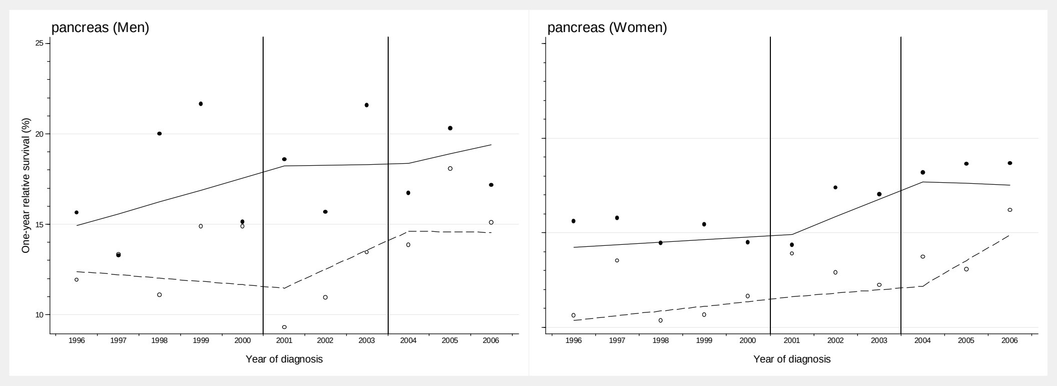
pancreas (Men)
pancreas (Women)
One-year relative survival (%)
Year of diagnosis
25
20
15
10
1996
1997
1998
1999
2000
2001
2002
2003
2004
2005
2006

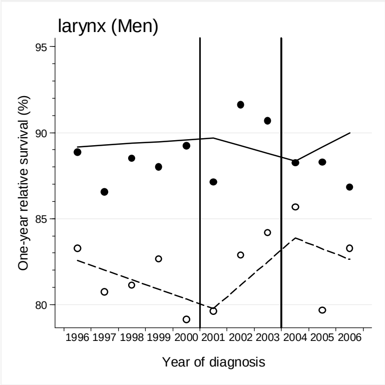
larynx (Men)
One-year relative survival (%)
Year of diagnosis
95
90
85
80
1996
1997
1998
1999
2000
2001
2002
2003
2004
2005
2006

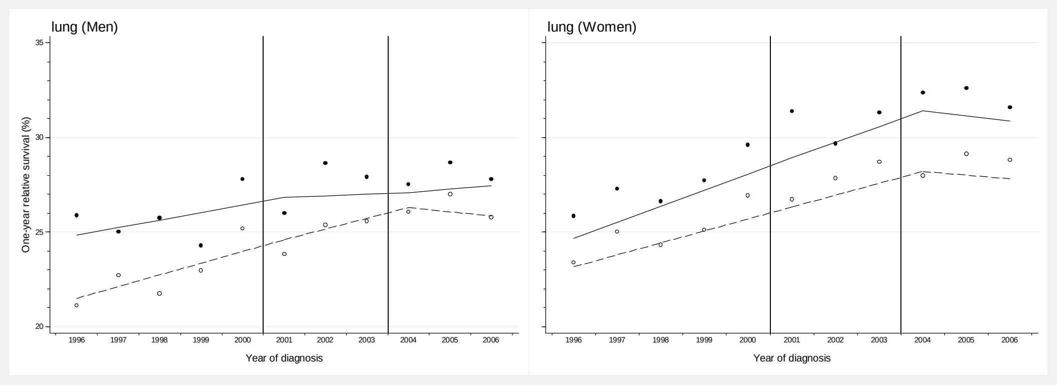
lung (Men)
lung (Women)
One-year relative survival (%)
Year of diagnosis
35
30
25
20
1996
1997
1998
1999
2000
2001
2002
2003
2004
2005
2006

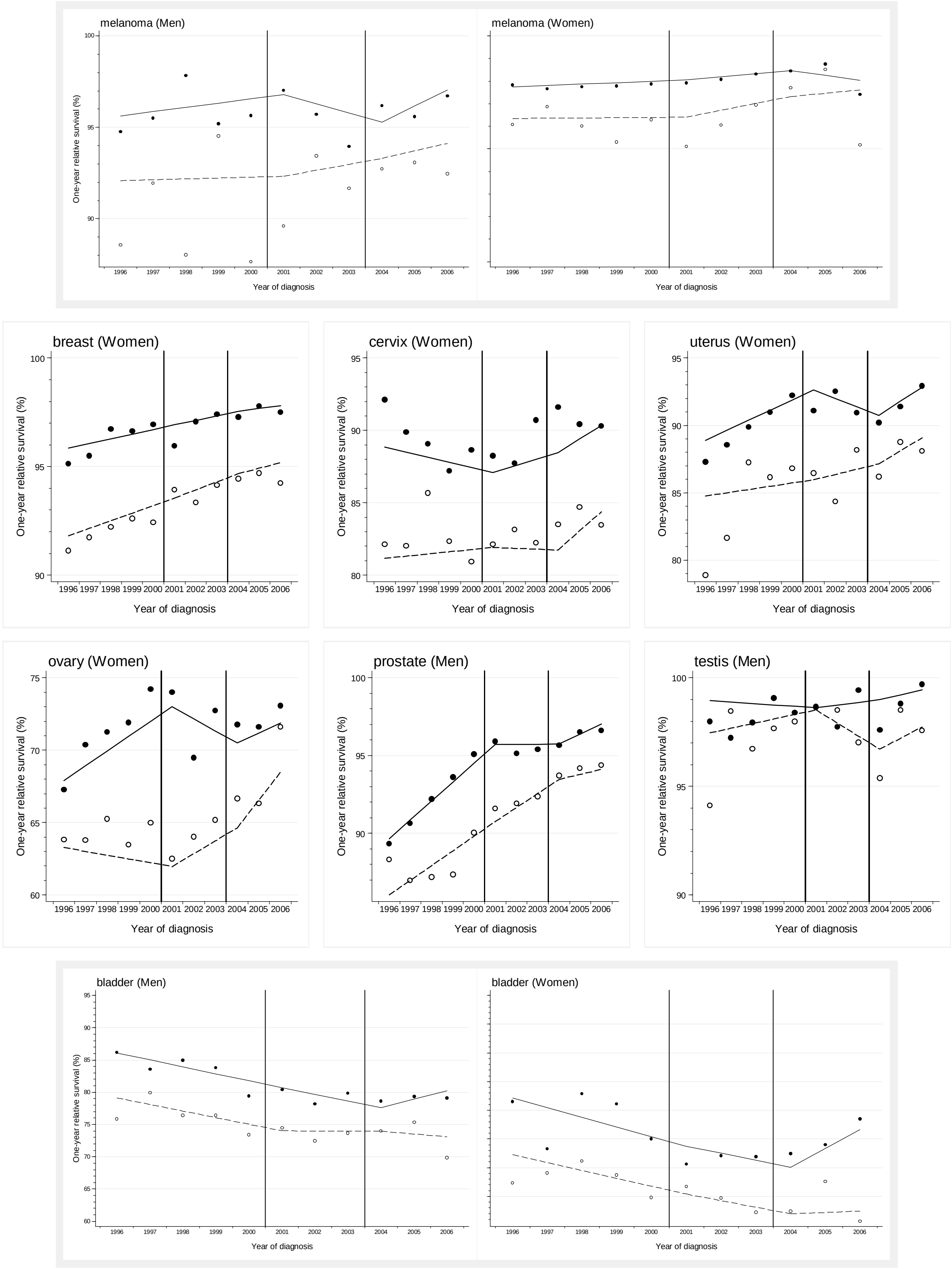
melanoma (Men)
melanoma (Women)
breast (Women)
cervix (Women)
uterus (Women)
ovary (Women)
prostate (Men)
testis (Men)
bladder (Men)
bladder (Women)
One-year relative survival (%)
Year of diagnosis
1996 1997 1998 1999 2000 2001 2002 2003 2004 2005 2006

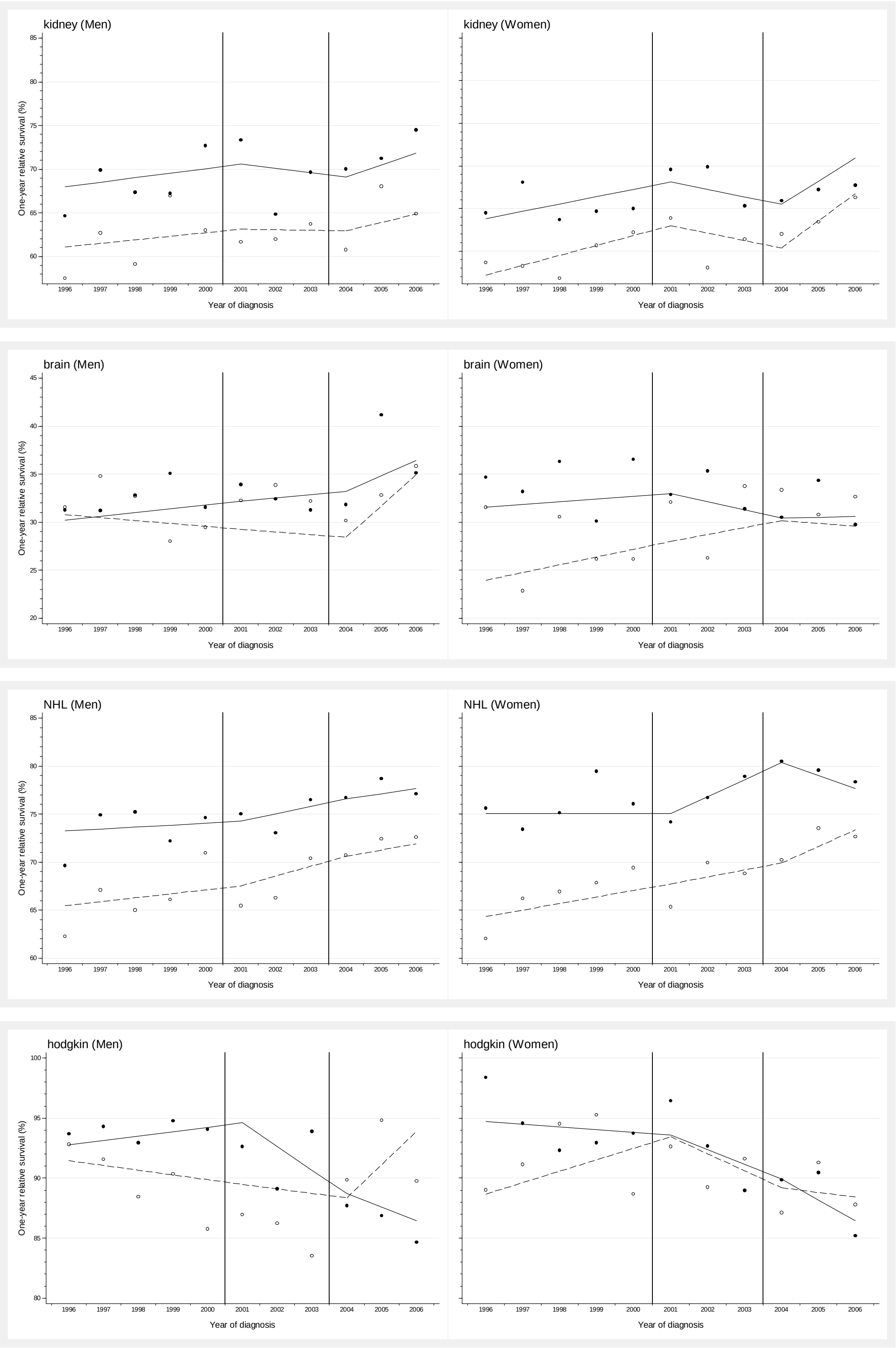
kidney (Men)
kidney (Women)
brain (Men)
brain (Women)
NHL (Men)
NHL (Women)
hodgkin (Men)
hodgkin (Women)
One-year relative survival (%)
Year of diagnosis
1996
1997
1998
1999
2000
2001
2002
2003
2004
2005
2006

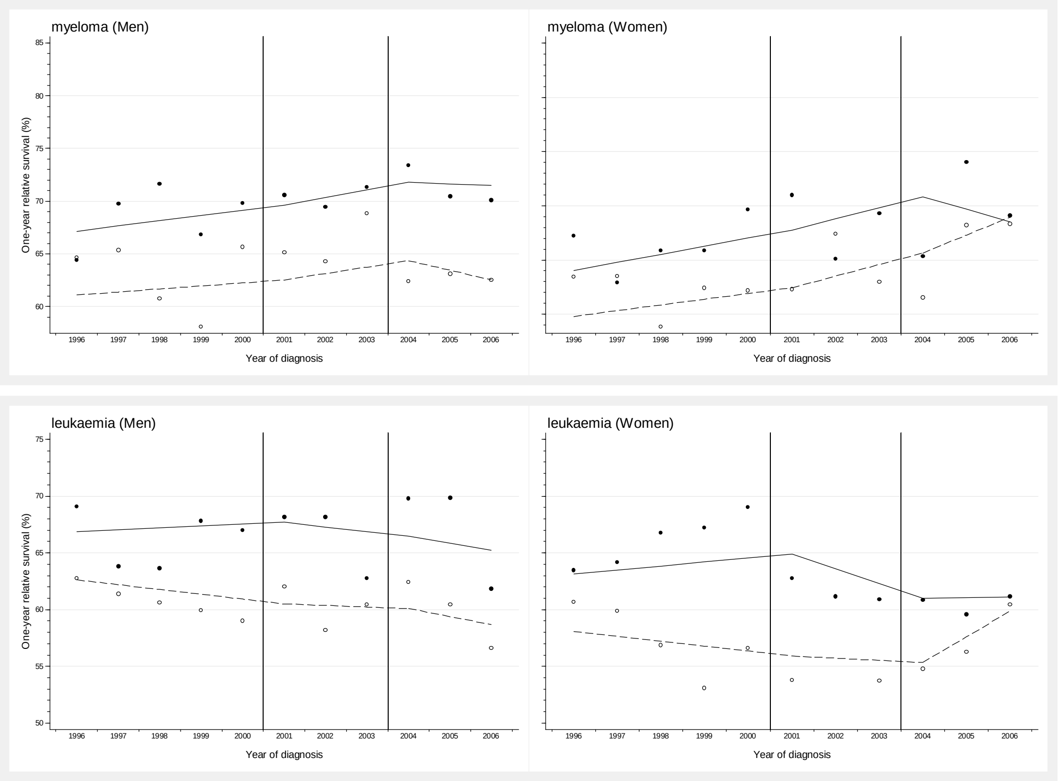

myeloma (Men)
myeloma (Women)
leukaemia (Men)
leukaemia (Women)
One-year relative survival (%)
Year of diagnosis
1996
1997
1998
1999
2000
2001
2002
2003
2004
2005
2006

Table 2. Deprivation gap in three-year relative survival (%) by sex and period of diagnosis, and change in the deprivation gap in survival, adults (15-99 years) diagnosed 1996-2006 and followed up to 2007: 21 common cancers, England

	Calendar period of diagnosis (NHS Cancer Plan periodisation)						Change in deprivation gap since previous period[b]				2007 (predicted[c])
	Period 1 1996-2000 (Pre-Cancer Plan)		Period 2 2001-2003 (Initialisation)		Period 3 2004-2006 (Implementation)						
Malignancy	Survival in most affluent	Deprivation gap (%)[a]	Survival in most affluent	Deprivation gap (%)[a]	Survival in most affluent	Deprivation gap (%)[a]	Period 2	95% CI	Period 3	95% CI	Deprivation gap (%)[a]
Oesophagus											
Men	12.0	-3.7 **	15.5	-5.1 **	16.7	-4.3 **	-1.3	-3.8 , 1.1	0.7	-2.5 , 3.8	-4.8 *
Women	11.4	-3.4 **	13.6	-2.3	15.7	-3.5 *	1.0	-2.2 , 4.2	-1.1	-5.3 , 3.1	-5.2 *
Stomach											
Men	17.5	-2.4 *	19.6	-3.8 **	19.2	-0.4	-1.4	-3.9 , 1.2	3.3	0.0 , 6.7	0.2
Women	18.6	-3.6 **	17.9	0.2	18.0	-1.4	3.8 *	0.3 , 7.4	-1.4	-5.9 , 3.2	-1.4
Colon											
Men	55.0	-6.6 **	56.0	-6.5 **	59.8	-8.5 **	0.1	-2.5 , 2.6	-2.0	-5.1 , 1.0	-8.9
Women	54.3	-7.4 **	55.2	-6.5 **	58.0	-8.8 **	0.9	-1.7 , 3.5	-2.2	-5.2 , 0.9	-9.1
Rectum											
Men	60.5	-12.1 **	62.2	-11.9 **	64.9	-13.0 **	0.1	-2.8 , 3.1	-1.1	-4.5 , 2.4	-10.9 **
Women	60.6	-9.3 **	62.2	-10.8 **	63.6	-9.0 **	-1.5	-5.2 , 2.1	1.7	-2.7 , 6.0	-12.0 **
Pancreas											
Men	4.3	-1.4 *	4.5	-1.6 *	5.3	-1.6	-0.3	-2.0 , 1.5	0.0	-2.2 , 2.3	-3.0 *
Women	3.5	-0.4	4.1	-0.8	3.6	1.0	-0.4	-2.0 , 1.2	1.8	-0.3 , 3.9	2.1
Larynx											
Men	75.2	-13.5 **	74.6	-11.9 **	76.6	-11.2 **	1.6	-4.1 , 7.3	0.8	-5.9 , 7.5	-10.6 *
Lung											
Men	9.2	-1.5 **	9.6	-1.2 *	10.0	-1.1 *	0.2	-0.8 , 1.2	0.2	-1.1 , 1.4	-1.8 *
Women	10.1	-1.3 *	12.2	-2.2 **	11.7	-1.0	-0.8	-2.2 , 0.5	1.1	-0.5 , 2.8	0.0
Melanoma											
Men	85.1	-6.7 **	87.0	-5.9 **	87.7	-6.9 **	0.8	-3.0 , 4.5	-1.0	-5.0 , 3.0	-8.2 **
Women	93.1	-3.7 **	93.8	-3.0 *	94.2	-1.6	0.7	-1.8 , 3.2	1.5	-1.2 , 4.2	-0.6
Breast											
Women	87.7	-6.7 **	89.6	-5.8 **	91.0	-4.7 **	0.8	-0.1 , 1.8	1.2 *	0.1 , 2.2	-4.0 **
Cervix											
Women	73.7	-7.7 **	76.0	-10.3 **	77.6	-9.0 **	-2.6	-6.6 , 1.4	1.1	-3.7 , 5.8	-9.4 **
Uterus											
Women	80.0	-5.7 **	81.5	-5.0 **	82.6	-6.2 **	0.7	-2.1 , 3.6	-1.1	-4.4 , 2.1	-5.9 **
Ovary											
Women	46.5	-3.1 *	47.8	-4.4 **	47.9	1.0	-1.3	-4.4 , 1.7	5.3 *	1.6 , 9.0	1.7
Prostate											
Men	80.1	-7.3 **	87.2	-6.9 **	89.8	-4.5 **	0.5	-1.0 , 1.9	2.3 *	0.9 , 3.8	-4.0 **
Testis											
Men	97.1	-1.4 *	97.4	-1.6	101.7	-9.4 *	-0.2	-2.3 , 1.9	-7.8 *	-15.4 , -0.3	-13.0 *
Kidney											
Men	54.6	-8.0 **	53.9	-6.5 **	56.0	-7.1 **	1.6	-2.6 , 5.8	-0.5	-5.2 , 4.2	-8.6 *
Women	51.2	-4.3 *	54.2	-5.9 *	55.4	-4.3	-1.6	-7.0 , 3.8	1.5	-4.6 , 7.5	-3.6
Bladder											
Men	70.7	-7.9 **	63.1	-5.9 **	61.7	-5.6 **	2.0	-1.0 , 5.0	0.4	-3.4 , 4.2	-7.7
Women	60.2	-10.1 **	50.4	-8.6 **	52.6	-13.4 **	1.5	-3.3 , 6.2	-4.7	-10.7 , 1.2	-19.1
Brain											
Men	15.7	2.8 *	14.7	4.3 *	16.0	0.8	1.5	-2.2 , 5.2	-3.4	-7.9 , 1.0	1.6 **
Women	17.6	-2.8 *	14.7	1.9	17.0	-0.5	4.7 *	0.2 , 9.2	-2.1	-7.5 , 3.3	-0.4 **
Hodgkin's disease											
Men	88.3	-6.0 *	85.5	-3.1	80.2	7.3 *	2.9	-3.5 , 9.2	10.6 *	3.0 , 18.2	6.3 *
Women	88.2	-5.6 *	87.7	-4.4	81.9	1.3	1.2	-5.9 , 8.3	5.7	-2.7 , 14.2	3.6
Non-Hodgkin lymphoma											
Men	59.1	-6.4 **	61.5	-6.8 **	65.3	-5.7 **	-0.4	-4.1 , 3.2	1.1	-3.0 , 5.2	-6.5 **
Women	61.3	-8.5 **	64.6	-9.3 **	68.1	-7.2 **	-0.8	-4.6 , 3.0	2.0	-2.3 , 6.3	-3.7 **
Myeloma											
Men	44.0	-8.5 **	43.5	-1.6	49.4	-8.3 *	6.9 *	1.0 , 12.8	-6.3	-13.3 , 0.8	-4.9
Women	41.4	-6.3 *	43.4	-5.0 *	47.5	-7.9 *	1.3	-5.0 , 7.5	-2.8	-10.5 , 5.0	-4.8
Leukaemia											
Men	51.6	-7.0 **	51.5	-7.0 **	51.5	-6.8 **	0.0	-4.2 , 4.3	0.2	-4.8 , 5.1	-7.4 *
Women	47.9	-6.5 **	47.5	-5.3 *	48.0	-4.8 *	1.3	-3.6 , 6.2	0.6	-5.2 , 6.4	-3.1

* p<0.05; **p<0.001

[a] Absolute difference (%) between relative survival in the most affluent and most deprived groups, derived from variance-weighted regression. A negative value means that survival in the most deprived group is lower than survival in the most affluent group

[b] Mean absolute change (%) in the deprivation gap in survival since the previous period. A neqative value means that the deprivation gap has widened

[c] Survival predicted for patients diagnosed in 2007 using the hybrid approach (see text)
